# Links Between Best-Friendship Quality and Well-Being From Early Emerging Adulthood to Early Established Adulthood

**DOI:** 10.1177/21676968241248877

**Published:** 2024-04-25

**Authors:** Stéphanie Langheit, François Poulin

**Affiliations:** 1 Department of Psychology, 14845 Université du Québec à Montréal, Montreal, QC, Canada

**Keywords:** friendship quality, emerging adulthood, well-being, romantic relationship, gender, loneliness, self-esteem

## Abstract

The aim of this study was to verify whether the links between features of best-friendship quality (intimacy, reliable alliance, conflict) and well-being indicators (self-esteem, loneliness) change from early emerging adulthood to early established adulthood. The moderating effect of gender and investment in romantic life on these links was examined as well. For the purpose, 346 individuals (58% women) completed questionnaires at age 20 and again at age 30. Multilevel analysis were performed for each well-being indicators separately. The results showed reliable alliance to be associated with both well-being indicators, and intimacy to be associated with loneliness. Age moderated the effect of intimacy on self-esteem, whereas investment in romantic life moderated the effect of reliable alliance. Finally, triple interactions emerged between conflict, gender and age in their associations with self-esteem and loneliness, underscoring particularities for men. These results underscore the most influential features of friendship quality for well-being.

## Introduction

Emerging adulthood is the period in life from ages 18 to 29 characterized, on the one hand, by favorable opportunities for positive development and, on the other, by demographic and social instability, risky behaviors, and developmental challenges likely of affecting well-being (Arnett, 2015; Nelson & Padilla-Walker, 2013). Cultivating quality friendships has been found to help in meeting the challenges of this period (Birditt & Antonucci, 2007; Cable et al., 2013; Crabtree, 2021; Schnyders et al., 2018). In fact, emerging adults turn to their friends when they feel distressed (Markiewicz et al., 2006; Stepleton et al., 2019; Trinke & Bartholomew, 1997). However, studies in this regard have been cross-sectional in design for the most part and are limited to samples of university students (approximately 19–22 years of age). As it happens, best-friendship quality changes from ages 19 to 30 (Langheit & Poulin, 2022). A best friend’s contribution to well-being might also change over this period (Pinquart & Sörensen, 2000). In established adulthood, individuals acquire greater stability but must also assume more responsibilities, particularly at the family and professional levels (Mehta et al., 2020). This can affect the quality of the best friendship and thus reduce its contribution to well-being (Demir et al., 2015). Finally, the link between friendship quality and well-being has been found to vary by gender (Lee et al., 2020; Miething et al., 2016; Wang et al., 2014). For these reasons, this study examined this link longitudinally from early emerging adulthood to early established adulthood, taking into account gender and investment in romantic life (Demir et al., 2018).

### Friendship and Well-Being

According to Deci and Ryan (2008), well-being refers to optimal functioning and an optimal psychological experience. It is a multidimensional concept that covers emotional, psychological, and social well-being. The first two refer to two major currents of thought: hedonic (pursuit of happiness and pleasure) and eudemonic (positive functioning and self-realization; Deci et al., 2008; Ryff, 1989), whereas the third, which has been rather neglected in the past, underscores the role of interpersonal relationships in functioning (Keyes, 2006). Social relationships are central in people’s well-being (Caunt et al., 2013). However, most research to date has focused primarly on the romantic relationship of emerging adults, neglecting other types. Yet, because it is egalitarian and voluntary, friendship is known to benefit these three facets of well-being (Froneman, 2014; Pezirkianidis et al., 2023). According to Need Fulfillment Theory, friends, like other social relations, serve to meet distinct social needs (Weiss, 1974). Furthermore, friends act as confidents and companions in addition to offering help and support that can be very usefull while going through the life transitions of emerging adulthood (Stress buffering model; Cohen & Wills, 1985; Wrzus et al., 2017). Friendship quality (i.e., the more *qualitative* characteristics of friendship) has been found to influence well-being more than do the more *quantitative* ones (e.g., contact frequency, duration; Antonucci, 2001; Demir, 2010). Friendship quality covers various features usually grouped under a positive dimension (e.g., intimacy, support) and a negative dimension (e.g., conflict; Bukowski et al., 1994; Demir et al., 2007; Ponti et al., 2010). The present study focused on three specific features of quality (intimacy, reliable alliance, and conflict) for their central importance in the definition of friendship and key functions in this relationship, as well as to measure both the positive and negative valence of friendship (Adams et al., 2000; Burke, 2023; Ponti et al., 2010). Langheit and Poulin (2022) also observed that these features changed during the course of emerging adulthood each in their own way. Best friendships stand above other less-close friendships across all of the quality features (Davis & Todd, 1985; Fehr, 1996; Hays, 1988). Moreover, the best friend is known to better satisfy social needs compared to other friendships (Demir et al., 2007).

Whereas the links between friendship quality and emotional well-being in emerging adulthood have been the focus of some studies (see research on happiness by Demir & Sümer, 2018), the psychological and social dimensions of well-being have been rather neglected. Self-esteem (psychological dimension) and loneliness (social dimension) are particularly salient indicators of well-being in emerging adulthood (Galambos et al., 2018; Qualter et al., 2015).

Where self-esteem is concerned, a review of the literature shows it to be positively associated with the positive dimension of friendship quality in emerging adults (Bagwell et al., 2005; Pittman & Richmond, 2008; Sherman et al., 2006). Some studies have reported a negative association between the negative dimension of quality and self-esteem (Sherman et al., 2006), while others observed no association at all (Bagwell et al., 2005). Finally, studies that examined more specific features of friendship quality noted that reliable alliance was associated with eudemonic well-being among emerging adults but that this was not the case for companionship and help (Anderson & Fowers, 2020).

Regarding loneliness in emerging adulthood, studies have reported a negative association with the positive dimension of friendship quality (Holt et al., 2018; Juvonen et al., 2022; Lee & Goldstein, 2016; Pittman & Richmond, 2008; Sherman et al., 2006) and a positive association with the negative dimension (Sherman et al., 2006). Two studies focused on specific features of friendship quality. Rook (1987) showed that intimacy (self-disclosure) was linked to a lower level of loneliness, whereas Heinrich and Gullone (2006) reported that conflicts with a friend were associated with a higher sense of loneliness. However, the reviewed research examined the links between friendship quality and well-being cross-sectionally and did not allow determining whether the best friend’s influence on well-being fluctuated over the course of emerging and established adulthood.

### Change in Links Between Friendship Quality and Well-Being

Emerging adulthood is characterized by the exploration of identity, romantic and professional possibilities (Arnett, 2015; Nelson, 2021). Emerging adults still do not necessarily assume all of the responsibilities of adulthood, such as financial independence, parenthood, and the pursuit of a career (Arnett, 2015). The instability that many experience, especially in early emerging adulthood, can generate considerable questioning and uncertainty that can affect their well-being (Arnett & Schwab, 2012; Lanctot & Poulin, 2018). In this regard, both self-esteem and loneliness have been found to increase over the course of emerging adulthood (Galambos et al., 2006; Qualter et al., 2015; Wagner et al., 2013).

The early 30s are marked by the start of a new phase of adult development that Mehta et al. (2020) have named *established adulthood* or that Knecht and Freund (2016) also refer to as the “rush hour of life.” During this time, individuals commit more to their occupation, gainning stability and advancement in their career. Commitment to family life also increases with more adults having their first child. It is to be noted that, as for emerging adulthood, established adulthood is not a homogenous period with a strict time frame, but mostly influenced by sociocultural processes (Mehta et al., 2020). Still, work and family life take up more space and comes with a lot of obligations reducing the time and energy invested in friendships (Hartup & Stevens, 1997). Nevertheless, Schmidt et al. (2023) reported that friendship quality remains positively associated with life satisfaction and negatively associated with loneliness at this period. Craig and Kuykendall (2019) also observed similar results for self-esteem.

From a life course perspective, developmental transitions can generate stress and bring about change in the composition of the support network (Carmichael et al., 2015; Elder Jr, 1998; Manalel & Antonucci, 2022). According to the Convoy model (Kahn & Antonucci, 1980), social relations are classified hierarchically in three concentric circles of closeness: *close*, *closer*, and *closest*. These convoys help navigate obstacles in life and bring support, guidance and protection (Antonucci et al., 2011). The *closest* relations have the greatest impact on well-being. While family relations (parents, siblings) generally fall within the circle of closest relations, friendships grow in importance from adolescence to early emerging adulthood before being gradually replaced by romantic relationships (Arnett, 2015; Markiewicz & Doyle, 2011). In fact, a best friend can eventually fall in the closest circle in emerging adulthood, when people do not live with their parents anymore and are not romantically engaged yet. Under this model, the link between friendship quality and well-being should diminish over the course of emerging adulthood. In support of this assumption, Miething et al. (2016) reported a significant decrease in the association between friendship quality and psychological well-being from ages 19 to 23. This decrease could be even more pronounced when individuals reach established adulthood (age 30). Finally, these links could be moderated by gender and investment in romantic life.

### Gender Differences

Studies that examined the effect of gender on the link between friendship quality and well-being have reported contradictory results. Several studies found friendship quality to be associated with well-being more among women (Almquist et al., 2014; Kaufman, 2020; Lee & Goldstein, 2016), but some found this association to be equal among men and women (Miething et al., 2016). Various factors, such as a lack of precision in the measurement of well-being and the choice of friendship quality dimensions might explain these discrepancies. In fact, Kauffman (2020) found that enjoyment was more strongly associated with well-being for woman then for men compared to closeness that was as important for both gender. Nevertheless, women ascribe greater importance to their friendships (Wang et al., 2014; Zhang et al., 2015) and give and receive more support than men do (Liebler & Sandefur, 2002). Moreover, some features of friendship more present among women (emotional support, self-disclosure, maintenance behaviors) have been found to be particularly beneficial to their well-being (Ryle, 2011). This could explain why they seem to benefit more from the positive dimension of friendship quality (Almquist et al., 2014; Kaufman, 2020). However, women have also been found to be more severely affected by the negative aspects of friendship (conflict, dissolution, emotional contagion; Lee & Goldstein, 2016; Sifers, 2011; Walen & Lachman, 2000). In sum, distinguishing the features of friendship and targeting more precise dimensions of well-being might allow clarifying the moderating effect of gender.

### Investment in Romantic Life

The link between friendship quality and well-being could also vary by investment in romantic life, given that romantic relationships themselves have a beneficial effect on well-being (Baumeister & Leary, 1995). This concept refers to the progressive stages of romantic roles and parenthood as conceptualized in past studies (Carbery & Buhrmester, 1998; Kalmijn, 2003). It can be operationalized as four stages in the following sequence: singlehood; having a romantic partner; living together - married or not - without children; and living together with children (Langheit & Poulin, 2022). According to Erikson (1968), establishing intimate relationships is the main developmental task of emerging adulthood. The need for intimacy in this period is met primarily by romantic partners, but friends can play a role in this regard as well. Indeed, the Diadic Withdrawal Hypothesis proposes that individuals withdraw from their friends to satisfy their needs for proximity with the romantic partner (Kalmijn, 2003; Milardo et al., 1983). However, as pointed out by Furman and Rose (2015), the effects that these two relationships have on well-being has rarely been explored simultaneously. The few studies that have done so, however, yielded mixed results; the link between friendship and well-being emerged as the most important of the two, persisted or, in some cases, disappeared when romantic relationships were taken into account (Bertera, 2005; Camirand & Poulin, 2022; Demir, 2010; Eshbaugh, 2010; Walen & Lachman, 2000). However, most of them observed that friendship quality stayed associatied with well-being even when other social relationships were included (partner and family; Bertera, 2005; Eshbaugh, 2010; Walen & Lachman, 2000). More studies are needed to specify these associations.

### The Present Study

In this longitudinal study, best-friendship quality, well-being and investment in romantic life were assessed in early emerging adulthood (age 20) and in early established adulthood (age 30). The purpose of the study was threefold. First, we sought to examine the links between best-friendship quality and well-being. Friendship quality was operationalized by taking account of the respective effects of two positive features—intimacy and reliable alliance—and one negative feature—conflict; and well-being was examined by distinguishing the psychological dimension—self-esteem—from the social dimension—loneliness. Our first hypothesis (H1) was that conflict would be associated positively with loneliness and negatively with self-esteem, whereas intimacy and reliable alliance would be associated positively with self-esteem and negatively with loneliness regardless of age (Bagwell et al., 2005; Newsom et al., 2003; Rook, 1990; Sherman et al., 2006).

Our second objective was to verify whether the links between best-friendship quality and well-being changed from early emerging adulthood to early established adulthood. We hypothesized (H2) that the links between friendship quality and well-being mentioned in our first hypothesis would be stronger in early ermerging adulthood than early established adulthood (Miething et al., 2016).

Our third objective was to test the moderating effect of gender on the link between best-friendship quality and well-being. We hypothesized (H3) that the links mentioned in H1 between, on the one hand, conflict and intimacy and, on the other, the two well-being variables would be stronger for women than for men (Almquist et al., 2014; Kaufman, 2020; Lee & Goldstein, 2016). No gender differences were expected regarding reliable alliance.

Our fourth objective was to test the moderating effect of investment in romantic life on the link between best-friendship quality and well-being. Based on Erikson’s theory, we hypothesized (H4) that all the links mentioned in H1 would be stronger for individuals less invested in their romantic life (Bertera, 2005; Demir et al., 2015). Lastly, the respective moderating effects of gender and investment in romantic life were examined by age (i.e, early emerging adulthood vs. early established adulthood) in an exploratory manner.

## Method

### Participants

To test these hypothesis, we used the data from a larger longitudinal study on social development from early adolescence to adulthood. The present report builds on our previous work with the same sample on change in friendship quality during emerging adulthood (Langheit & Poulin, 2022). This longitudinal study was initiated in 2001 and originally included 390 sixth-graders (58% girls, *M* = 12.38 years, *SD* = 0.42) from eight schools in a suburban area north of Montreal (Canada). Approximately 75% of the available student population participated in this study. The vast majority (90%) of the sample were Caucasian; the others were Black (3%), Hispanic (3%), Arab (3%), and Asian (1%). At the first data collection time point, 72% of the participants lived with their two biological parents and the mean family income ranged from $45,000 to $55,000. Data were then collected from the sample periodically until age 30. For the purposes of our study, we used data collected in 2010 (*M* = 20.17 years, *SD* = 0.43, min = 18.86, max = 21.55) and in 2020 (*M* = 30.25 years, *SD* = 0.41, min = 30.07, max = 31.54). The present subsample consisted of participants from whom data were collected at one or both of these time points. Compared with the rest of initial sample (*n* = 44), the retained participants (*n* = 346) included a larger proportion of women (60,5% vs. 39,5%; chi-square test = 6.93 *p* < .01) and were more likely to come from a nuclear family with their two biological parents (72,6% vs. 39,5%, chi-square test = 20.83 *p* < .001), but did not differ significantly on family income and ethnic background.

### Procedure

This longitudinal sample initially was recruited in Grade 6 following three steps. First, the project was presented to the school officials and Grade 6 teachers who agreed to be part of the study. Second, the project was described to the Grade 6 students in class by graduate research assistants. Third, the students who were interested in the project were asked to bring home to their parents a flyer and a consent form. Only the students who brought back the consent form signed by their parents were part of the study. All of the variables were measured with the same instruments at ages 20 and 30. At age 20, a research assistant visited participants at home where they completed a questionnaire. A few participants (less than 5%) received their questionnaire by mail with a postage-paid return envelope. At age 30, participants completed the questionnaire online. At both time points, participants provided written consent and received financial compensation. The study was approved by the Research Ethics Board of the Université du Québec à Montréal.

### Measures at Ages 20 and 30

#### Best-Friendship Quality

First, participants were asked to write down the name of the person they considered their best friend (first and last name). They were told that this person could not be a romantic partner or a family member. The majority of the best friends thus designated were of the same gender as the respondent (85% at 20; 92.8% at 30). Most participants (73.6%) named a different friend at age 20 and age 30.

Second, participants had to answer a series of questions on this friendship. The items were taken from the *Network of Relationships Inventory* (NRI) developed by Furman and Buhrmester (1985). Three items measured intimacy (e.g., *How often do you share secrets and private feelings with this person?*); three items measured reliable alliance (e.g., *How sure are you that this relationship will last no matter what?*); and three items measured conflict (e.g., *How often do you and this person argue with each other?*). Participants had to rate how much they agreed with each item on a five-point Likert scale from 1, v*ery little or none of the time*, to 5, *most of the time*. Internal consistency (Omega) at 20 and 30 was .84 and .84 for intimacy, .93 and .95 for reliable alliance, and .75 and .66 for conflict. The NRI has been shown to have good predictive, factor and construct validity (Furman, 1996).

#### Self-Esteem

Participants had to indicate how much they agreed with each of the 10 items (e.g., *On the whole, I am satisfied with myself*) of the *Rosenberg Self-Esteem Scale* (Rosenberg, 1965) on a four-point Likert scale from *strongly agree* to *strongly disagree*. The degree of self-esteem corresponds to the sum of the scores for the 10 items. The higher the score, the higher the self-esteem. The instrument has been shown to possess good reliability and validity (Rosenberg, 1965). Its internal consistency was excellent with Omega's of .9 at 20 and .94 at 30 .

#### Loneliness

We used ten items from the *UCLA Loneliness Scale* (Russell et al., 1980) to measure sense of loneliness. Participants had to indicate how often each of the items (e.g., *I feel completely alone*) was descriptive of them on a four-point Likert scale from *often* to *never*. The degree of loneliness corresponded to the sum of the scores on the 10 items. The higher the score, the higher the loneliness. The instrument’s validity has been demonstrated across a broad range of ages and populations (Russell, 1996). The scale showed good internal coherence, obtaining Omega’s of .89 at 20 and .94 at 30.

#### Investment in Romantic Life

Participants had to indicate: (1) whether they had a romantic partner (yes/no); (2) whether they were living with the person (yes/no); and (3) whether they had children (yes/no). An investment in romantic life variable was then created at ages 20 and 30 based on this information. This was a four-level variable that was treated in the analyses as a continuous variable: (0) single; (1) with romantic partner but not living together and without children; (2) living with romantic partner but without children; and (3) living with romantic partner and with children. Two unconventional patterns emerged in our data: with romantic partner and children but not living together and single but with children. To specifically target investment in romantic life, these cases were coded 1 (“with romantic partner”) and 0 (“single”), respectively. Only 10 participants fit either one of these patterns at either one of the data collection time points. This operationalization of investment in romantic life was informed by the work of Carbery and Buhrmester (1998) and Kalmijn (2003).

### Data Analysis Strategy

Multilevel analysis was employed to achieve the study’s four objectives. Separate models were tested for self-esteem and loneliness. The independent variables included in the models were the following: (1) three features of best-friendship quality (intimacy, reliable alliance and conflict); (2) age; (3) gender; (4) investment in romantic life; (5) interactions between each feature and age; (6) interactions between each feature and one of the two moderator variables (gender or investment in romantic life); and (7) triple interactions between each feature, one of the two moderator variables (gender or investment in romantic life) and age.

The analysis followed the model below when gender was used as a moderator:

#### Level 1

*Y*_*ij*_ = *β*_*0i*_ + *β*_*1i*_*(*intimacy_ij_*)* + *β*_*2i*_*(*reliable alliance_ij_*)* + *β*_*3i*_*(*conflict_ij_*)* + *β*_*4i*_*(*age_ij_*)* +* β*_*5i*_*(*romantic life_*ij*_*)* + *β*_*6i*_*(*age_ij_*intimacy_*ij*_*)* + *β*_*7i*_*(*age_ij_*reliable alliance_*ij*_) + *β*_*8i*_*(*age_ij_*conflict_*i*j_*)* + ε_*ij*_

#### Level 2

*β*_*0-*__*8*__*i*_ = *γ*_*00*_ + *γ*_*01*_(gender_*i*_) + *u*_0i_

The analysis followed the model below when investment in romantic life was used as a moderator:

#### Level 1

*Y*_*ij*_ = *β*_*0i*_ + *β*_*1i*_*(*intimacy_*ij*_*)* + *β*_*2i*_*(*reliable alliance_*ij*_*)* + *β*_*3i*_*(*conflict_*ij*_*)* + *β*_*4i*_*(*age_*ij*_*)* + *β*_*5i*_*(*romantic life_*ij*_*)* + *β*_*6i*_*(*age_ij_*intimacy_*ij*_*)* + *β*_*7i*_*(*age_ij_*reliable alliance_*ij*_*) *+ *β*_*8i*_*(*age_ij_*conflict_*ij*_*) *+ *β*_*9i*_*(*romantic life_ij_*intimacy_*ij*_*)* + *β*_*10i*_*(*romantic life_ij_*reliable alliance_*ij*_*)* + *β*_*11i*_(romantic life_ij_*conflict_*ij*_*)* + *β*_*12i*_*(*age_ij_*romantic life_ij_*intimacy_*ij*_) + *β*_*13i*_(age_ij_*romantic life_ij_*reliable alliance_*ij*_) + *β*_*14i*_*(*age_ij_*romantic_ij_*conflict_*ij*_) + ε_*ij*_

#### Level 2

*β_0-14__i_* = *γ*_*00*_ + *γ*_*0*_*1*__ + *u*_*0i*_.

First, the three features of best-friendship quality (intimacy, reliable alliance, conflict), age, gender, and investment in romantic life were entered in the model to examine their main effects (objective 1). Second, the interactions between the friendship features and age were added to the model to determine whether the effect of friendship quality varied by age (objective 2). Third, the interactions between gender and the features of friendship quality and the triple interactions between gender, the features of best-friendship quality and age were added to assess the moderating effect of gender on the links under study (objectives 3 and 4). Finally, this same model was examined with investment in romantic life replacing gender as the moderator (objectives 3 and 4). When an interaction was significant, the simple effects for each group were examined. The multilevel analysis function in R (lme) uses listwise deletion for every row of values with a missing observation and then re-adjust estimates with maximum likelihood.

## Results

### Preliminary and Descriptive Analyses

Table 1 presents the descriptive statistics for the three features of friendship quality and for the two indicators of well-being at ages 20 and 30. A logarithmic transformation was applied to the variables self-esteem and loneliness in order to fix their non-normal distribution. Table 2 presents the correlations among the variables. The table shows that, at age 20, the two well-being indicators were associated with all the features of friendship quality in the expected direction. At age 30, self-esteem was associated with reliable alliance only, whereas loneliness was associated with the two positive features (intimacy and reliable alliance), but not the negative one (conflict). Men reported higher self-esteem than women did, but the two did not differ in terms of loneliness. Women scored higher on intimacy at age 20 and at age 30 and scored lower on conflict at age 30 than men did. Investment in romantic life was positively associated with self-esteem at age 20 and negatively associated with loneliness at age 20 and at age 30. The features of friendship quality were not linked to investment in romantic life. Finally, as the correlations between the three features of friendship quality were significant, a multicollinearity test was carried out. All of the values of this test fell below the critical threshold of 2.5 (Pituch & Stevens, 2016).

**Table 1.** Descriptive Statistics for Friendship Quality Features, Well-Being Indicators, and Investment in Romantic Life at Ages 20 and 30.

	Age 20 (*n* = 304)				Age 30 (*n* = 322)			
	Min	Max	M	SD	Min	Max	M	SD
Intimacy	1.67	5.00	4.14	0.88	1.00	5.00	3.97	0.96
Reliable alliance	2.00	5.00	4.61	0.61	1.00	5.00	4.27	0.91
Conflict	1.00	4.33	1.62	0.64	1.00	5.00	1.37	0.54
Self-esteem	1.10	3.00	2.66	0.42	0.70	3.00	2.49	0.52
Loneliness	1.00	3.00	1.52	0.49	1.00	3.50	1.67	0.60
Invest romantic life	0.00	3.00	0.68	0.71	0.00	3.00	1.95	1.09

**Table 2.** Correlations Among Study Variables at Ages 20 and 30.

	1	2	3	4	5	6	7
1. Intimacy	1	.40***	−.06	.13*	−.23***	−.32***	−.00
2. Reliable alliance	.63***	1	−.07	.20***	−.22***	−.02	−.08
3. Conflict	−.04	−.11*	1	−.14*	.17**	.08	−.07
4. Self-esteem	.04	.15**	.03	1	−.53***	.15**	−.06
5. Loneliness	−.23***	−.34***	.05	−.59***	1	−.00	−.11*
6. Gender	−.17**	−.08	.20***	.13*	.02	1	−.23***
7. Invest romantic life	.04	.08	−.08	.13*	−.23***	−.15**	1

*Note.* Correlations at age 20 are presented above the diagonal, those at age 30 below it.

*p < .05. **p < .01. ***p < .001.

### Main Effects Analyses

#### Self-Esteem

Significant main effects on self-esteem were observed for reliable alliance, investment in romantic life, gender, and age. Specifically, high levels of reliable alliance, *β* = .15, *t*(266) = 3.32, *p =* .001, and investment in romantic life, *β* = .12, *t*(266) = 2.6, *p* = .01, were found to be associated with higher self-esteem. Moreover, men reported higher self-esteem than women did, *β* = .16, *t*(344) = 3.34, *p* = .001. Finally, self-esteem was higher at age 20 than at age 30, *β* = −.22, *t*(266) = −5.19, *p* < .001. No main effect emerged for conflict and intimacy.

**Figure 1.**
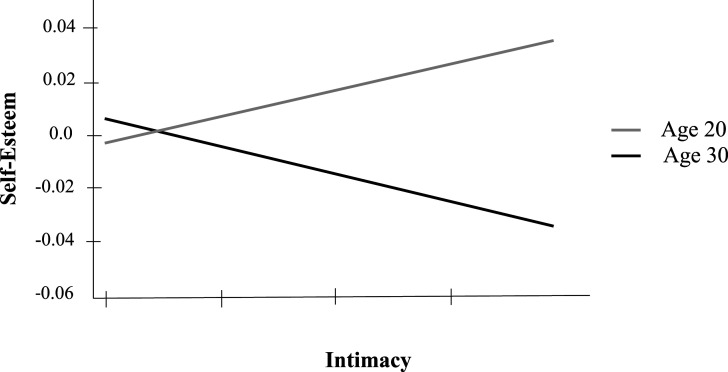
Self-esteem predicted by interaction between best-friendship intimacy and age.

Adding the interactions with age revealed the presence of an interaction between age and intimacy, *β* = −.08, *t*(264) = −2.24, *p* = .03. Results of simple-effect analysis showed a positive slope between intimacy and self-esteem at age 20, *β* = .13, *t*(289) = 2.11, *p* = .035, but no significant association at 30, *β* = −.07, *t*(316) = −.97, *p* = .335 (see Figure 1). Associations observed earlier remained significant and of similar strength (reliable alliance, age, investment in romantic life and gender).

**Figure 2.**
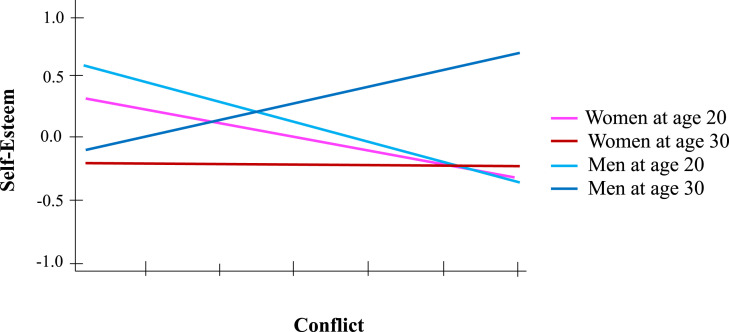
Self-esteem predicted by interaction between best-friendship conflict, age, and gender.

Adding the interactions with gender revealed a triple interaction between conflict, age, and gender, *β* = .1, *t*(256) = 2.66, *p* = .008, in addition to the other associations. Analyzing the simple two-way interaction effects yielded a significant interaction between age and conflict among men, *β* = .15, *t*(98) = 2.95, *p =* .004. This interaction was not significant among women, *β* = −.05, *t*(157) = −0.96, *p* = .34. In fact, conflict was not associated with self-esteem among women, *β* = −.05, *t*(157) = −.95, *p* = .34, regardless of age. However, among men, the presence of friendship conflict was associated with lower self-esteem at age 20, *β* = −.17, *t*(112) = −2.41, *p* = .018; while at age 30, it was associated with higher self-esteem, *β* = .17, *t*(120) = 1.99, *p* = .049 (see Figure 2).

Finally, adding the interactions with investment in romantic life (instead of those with gender) revealed an interaction between reliable alliance and investment in romantic life, *β* = −.16, *t*(256) = −2.48, *p* = .01, in addition to main effects and the intimacy by age interaction. The more committed participants were to a romantic relationship, the less the level of best-friend reliable alliance was associated with self-esteem, and this was the case regardless of age.

#### Loneliness

Main effects were observed for intimacy, reliable alliance, age, and investment in romantic life on loneliness. Specifically, high levels of intimacy, *β* = −.11, *t*(266) = −2.33, *p* = .02, reliable alliance, *β* = −.21, *t*(266) = −4.91, *p* < .001, and investment in romantic life, *β* = −.21, *t*(266) = −4.57, *p* < .001, were found to be associated with a lower sense of loneliness. Furthermore, loneliness was less pronounced at age 20 than at age 30, *β* = .2, *t*(266) = 4.79, *p* < .001. No main effect was observed for conflict and gender.

Adding the interactions with age yielded no other significant effect.

**Figure 3.**
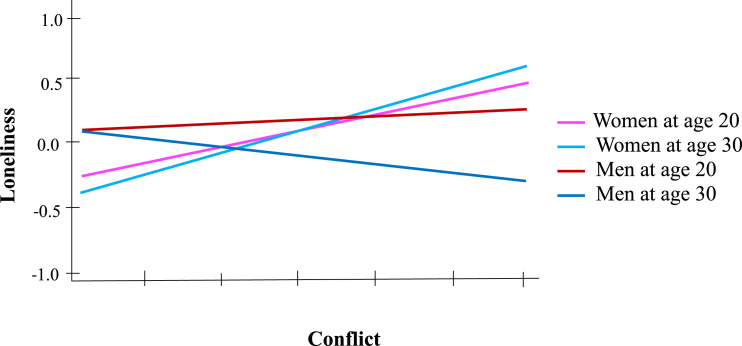
Loneliness predicted by interaction between best-friendship conflict, age, and gender.

Adding the interactions with gender revealed the presence of a triple interaction between conflict, age and gender, *β* = −.07, *t*(256) = −2.05, *p* = .041, in addition to the other main effects. Analyzing the simple two-way interaction effects revealed a significant interaction between age and conflict for men, *β* = −.13, *t*(98) = −2.72, *p =* .008, but the interaction failed to reach statistical significance for women, *β* = .02, *t*(157) = 0.41, *p* = .69. For women, conflict with the best-friend were not associated with loneliness, *β* = .08, *t*(157) = 1.43, *p* = .16, regardless of age. Simple-effect analysis brought to light a positive association between conflict and loneliness for men at age 20, *β* = .18, *t*(112) = 2.61, *p =* .023, but no significant association for men at age 30, *β* = −.1, *t*(120) = −1.24, *p* = .22 (see Figure 3). Finally, no significant effect was observed for interactions involving investment in romantic life.

## Discussion

Studies have evidenced the contribution of romantic relationships to the well-being of emerging adults (Gómez-López et al., 2019). However, the potential contribution of friendship has seldom been investigated in this age group (Markiewicz et al., 2006). This study looked at the links between three features of best-friendship quality—intimacy, reliable alliance and conflict—and two indicators of well-being—self-esteem and loneliness. Specifically, we examined how these links changed from early emerging adulthood to early established adulthood and whether gender and investment in romantic life had a moderating effect on these links. The results show the presence of distinct links between the features of best-friendship quality and the two well-being indicators with reliable alliance keeping it’s importance all through emerging adulthood. Moreover, links with conflict and intimacy change from early emerging adulthood to early established adulthood. Finally, gender and investment in romantic life have a moderating effect on conflict for the first and reliable alliance for the second. The patterns of results observed for self-esteem and loneliness present similarities and differences. These two dimensions of well-being will be addressed simultaneously in the discussion though they were analyzed in distinct models. Overall, although significant findings are observed, the effect sizes were relatively small. Thus best-friendship quality should be considered as one factor among several others to be associated with well-being in emerging adulthood.

### Links Between Best-Friendship Quality and Well-Being

Emerging adults who perceived higher levels of best-friendship reliable alliance reported higher self-esteem and less loneliness. These results confirm in part our first hypothesis and are consistent with previous studies that demonstrated that higher-quality friendships fostered the well-being of individuals (Bagwell et al., 2005; Craig & Kuykendall, 2019; Froneman, 2014). They also go a little further by specifying how reliable alliance is a key feature of friendship quality, as also pointed out by Anderson and Fowers (2020). In fact, as underlined in the stress buffering model, the simple perception of the friend’s availibility helps to re-evaluate the potential danger of a situation and the ability to cope with it (Cohen & Wills, 1985; Lee & Goldstein, 2016). Also, what distinguishes friendship from family and romantic relationships is that it is voluntary, egalitarian and more flexible because devoid of moral and legal obligations (Merz et al., 2009). In this regard, sense of reliable alliance attests to the perception that a friend cares enough about us (and reciprocally) and that we hold enough value in their eyes for them to choose to maintain the relationship despite obstacles. Hence, this finding is in line with the theory of self-determination, which stipulates that human beings establish their worth based on their interpersonal relationships and depend on them to achieve their goals (Anderson & Fowers, 2020; Baumeister & Leary, 1995; Leary, 2005). It also supports attachment theory, attesting how relationship security even with the best friend is fundamental for well-being (La Guardia et al., 2000).

Best-friend intimacy was also associated with less loneliness. This result also supports previous studies on links between intimacy and loneliness (Lee & Goldstein, 2016; Rook, 1987; Schmidt et al., 2023) and is consistent with those regarding fulfillment of the need for social integration, which friendship is believed to meet (i.e., need fulfillment; Carbery & Buhrmester, 1998; Deci et al., 2006).

Finally, contrary to expectations, conflict was not associated with self-esteem or loneliness (Bagwell et al., 2005; Newsom et al., 2003; Rook, 1990; Sherman et al., 2006). However, links did emerge when age and gender were taken into account.

### Change in Links from Early Emerging Adulthood to Early Established Adulthood

Our second hypothesis stipulated that these links would be stronger in early emerging adulthood than in early established adulthood (Arnett, 2023). The results support this hypothesis for intimacy and conflit (for men only; see below). As highlighted by the Convoy Model, individuals seem to experience a reorganization of their social network during emerging adulthood. Consequently, certain aspects of best-friendship quality become less closely associated with well-being (Kahn & Antonucci, 1980; Manalel & Antonucci, 2022; Miething et al., 2016). Indeed, the link between intimacy and self-esteem varied by age. At age 20, emerging adults who perceived more best-friend intimacy have higher self-esteem. At age 30, this link disapeared. The early 20s are characterized by identity exploration and best-friends are key actors in this process (Crabtree, 2021; Erikson, 1968; Parker & Gottman, 1989). Our results might suggest that self-disclosure with best-friend contributes to identity development in early emerging adulthood in particular, thereby fostering self-esteem. However, in early established adulthood, identity and self-esteem may be more stable and thus less likely to be influenced by levels of self-disclosure with friends. As people enter the “career and care crunch”, their self-esteem is possibly more closely linked to their professional life and their own family relationships (couple and parenting) and less to their best-friendship (Mehta et al., 2020).

Our results also suggest a certain degree of stability in how friendship quality, specifically reliable alliance, contributes to well-being from early emerging adulthood to early established adulthood, which is coherent with Schmidt and colleagues’ (2023) findings.

### Gender and Investment in Romantic Life

The third and fourth goals of the study were to test the moderating effect of gender and investment in romantic life on the link between best-friendship quality and well-being in general (double interaction) and by age (triple interaction). No double interaction turned out significant for gender, but an interaction with investment in romantic life and triple interactions were (see below).

First of all, the link between reliable alliance and self-esteem was moderated by investment in romantic life. The more invested emerging adults were in their romantic relationships, the less best-friend reliable alliance was associated with their self-esteem. This finding supports only in part our fourth hypothesis as it applies specifically to reliable alliance in friendship. This results could shed some light on the mixed results observed in the literature. It could be explained by the fact that individuals more invested in romantic life cultivate other significant stable relationships as well (romantic and/or children). By meeting the need for durable relationships, these other relationships, too, gratify the individual (Bertera, 2005; Demir et al., 2007; Erikson, 1968). Thus, their presence might diminish the impact of friendship on self-esteem. This result is also consistent with the Diadic Withdrawal Hypothesis stipulating that individuals engaged in a romantic relationship would gradually withdraw from their friends and satisfy their needs for proximity with the romantic partner (Milardo et al., 1983). The association between the other features of friendship and self-esteem did not vary by investment in romantic life. As mentionned above, according to the Need Fulfillment Theory, friendship seems to continue to satisfy certain social needs regardless of the presence of other interpersonal relationships (Eshbaugh, 2010; Furman & Buhrmester, 1992; Walen & Lachman, 2000; Weiss, 1974).

In addition, a triple interaction was observed between conflict, age, and gender for both self-esteem and loneliness. For men only, level of conflict was negatively associated with self-esteem and positively associated with loneliness in early emerging adulthood, but not in early established adulthood. Conflict could have a greater effect on men’s well-being given that their best-friendships are also less intimate, reducing the possibility of a compensatory effect. In fact, women tend to be more intimate and supportive with their friends than men are in addition of being less conflictual (Langheit & Poulin, 2022; Liebler & Sandefur, 2002). The positive features of women’s friendship could play a protective role in their well-being. Also, a stronger tendency for communal conflict resolution strategies could also allow women to better resolve their conflicts with best-friend reducing its impact on their self-esteem (Keener et al., 2012). This could explain why, for women, level of conflict was not associated with either self-esteem or loneliness, regardless of age. Moreover, Langheit and Poulin (2022) noted that, at least where intimacy is concerned, this gender difference faded in early established adulthood, which could explain why we observed this link only at age 20. Early emerging adulthood is also characterized by numerous demographic and social transitions (e.g., leaving the family home, changing school or employment) that can make it hard to maintain friendships and can increase one’s sense of loneliness (Qualter et al., 2015). Consequently, an increase, however small, in conflicts within a relationship as significant as the best-friendship could have a considerable effect on well-being during this period of vulnerability.

Yet, we cannot rule out the hypothesis that, in early emerging adulthood, men with low self-esteem and a high sense of loneliness tend to have more conflictual friendships (transactional model; Sameroff, 2009). In this regard, it is believed that socialization leads young men to repress their psychological distress instead of seeking help or expressing their anger, as these two options could generate conflicts (Bank & Hansford, 2000; Lips, 2018). This could also explain the positive association between levels of conflit and self-esteem for men at age 30. It is possible that with age and and a stronger self-esteem, men learn to better express their feelings making it possible to have conflict, but also resolve them.

Finally, the absence of gender differences for reliable alliance and intimacy is in line with research by Kaufman (2020) and Miething and colleagues (2016) suggesting a certain level of similarity between gender on the importance of closeness for well-being.

### Strengths, Limitations, and Future Research

The principal strength of this study is the use of a longitudinal design spanning 10 years and covering the early phase of emerging adulthood up to the beginning of established adulthood. This design allowed testing directly the effect of age on the links between friendship quality and well-being. Most of the research on the subject has been cross-sectional and compared groups of individuals of different ages, which did not allow analyzing intra-individual changes (Anderson & Fowers, 2020; Lee & Goldstein, 2016). Another strength lies in the simultaneous examination of the distinct effect of several features of friendship quality (intimacy, reliable alliance, and conflict). This allowed us to highlight the specificity of the associations and, above all, to sharpen our understanding of the associations between different aspects of friendship and well-being.

Our study is not without limitations. First, all the study variables were based on self-report measures, which can give rise to problems of shared method variance. Second, the best friend’s point of view on the existence of friendship was not taken into account. Though reciprocity is considered by many researchers to be a key criterion in identifying friendships (Hartup & Stevens, 1997), it is harder to apply in studies conducted with emerging adults. Along the same line, we would stand to gain from also taking account of the friend’s point of view on the quality of the relationship. Third, the reliability of the conflict scale was somewhat low at age 30. Low reliability created by ceiling and floor effects could indicate a lack of statistical power and limit the detections of significant associations when they are weak. Fourth, research would benefit from including other features of friendship quality such as instrumental and emotional support, admiration, antagonism, etc., that could differently be associated with well-being. Finally, our sample was rather homogenous. It could be said to be representative only of White suburban middle-class French-speaking Canadians. It is also important to remain cautious about generalization of the current findings as a larger portion of participants are women and come from nuclear family. Also, most of our participants reported a same-gender best friend and a romantic partner of the other gender. Besides, the examination of gender was entirely binary, although this concept is mostly viewed as a continuum. Consequently, further studies should be conducted to verify if the current findings hold for nonbinary individuals, mixed-gender friendships and same-gender couples. Gender and sexual orientation can have an influence on how friendship is conceived and on social norms (Muraco, 2012). On one hand, the adherence to gender roles was seen to be associated with friendship quality (Bowman, 2009). On the other hand, challenges faced by LGBTQ + population such as social and family exclusion, can influence their views of friendships and their quality (Shilo & Savaya, 2011). Finally, analyzing the joint effect of romantic relationship quality and friendship quality on well-being might allow us to arrive at a better understanding of the phenomenon. This study brings additional knowledge on the role of best-friendship in emerging adulthood and how it can promote well-being. Also, these results underline the importance that therapists assess friendship quality in psychotherapy and help emerging adults develop better capacities to create deep connections, share intimate thoughts and solve conflicts to improve their well-being.

## Conclusion

Our study showed the importance of reliable alliance in best-friendship for well-being, particularly for individuals less invested in romantic life. Some links between best-friendship quality and well-being varied by age, gender, and investment in romantic life. Best-friendship intimacy and conflict (for men) were associated with well-being in early emerging adulthood but waned in importance thereafter.

## Supplemental Material

Supplemental Material - Links Between Best-Friendship Quality and Well-Being From Early Emerging Adulthood to Early Established Adulthood Supplemental Material for Links Between Best-Friendship Quality and Well-Being From Early Emerging Adulthood to Early Established Adulthood by Stéphanie Langheit and Françcois Poulin in Emerging Adulthood
